# Large Intramural Aortic Hematoma with Intimal Tear

**DOI:** 10.1055/s-0040-1714058

**Published:** 2020-11-09

**Authors:** Frédéric Jacques, Michel Gingras, Valérie Lafrenière-Bessi, Jean Perron, François Dagenais

**Affiliations:** 1 Departments of Cardiac Surgery, Institut Universitaire de Cardiologie et de Pneumologie de Québec–IUCPQ, Quebec, QC, Canada; 2 Departments of Medical Imaging, Institut Universitaire de Cardiologie et de Pneumologie de Québec–IUCPQ, Quebec, QC, Canada

**Keywords:** aortic intramural hematoma, dissection, acute aortic syndrome

## Abstract

A 72-year-old man presented with excruciating epigastric pain. A chest computed tomography angiography revealed an aortic intramural hematoma. A filling defect within the distal ascending aorta was noted. Images of an intramular hematoma and surgical details of an ascending aortic replacement under deep hypothermic circulatory arrest are provided.

A 72-year-old man presented with excruciating epigastric pain not associated with exercise or trauma. After excluding myocardial ischemia, a chest computed tomography angiography revealed an aortic intramural hematoma (IMH) of 5 mm in thickness starting at the sinotubular junction (34 mm) and extending circumferentially to the diaphragm (27 mm; Fig. 1A–C ). A filling defect within the distal ascending aorta was present ( Fig. 1A–C ).

**Fig. 1:**
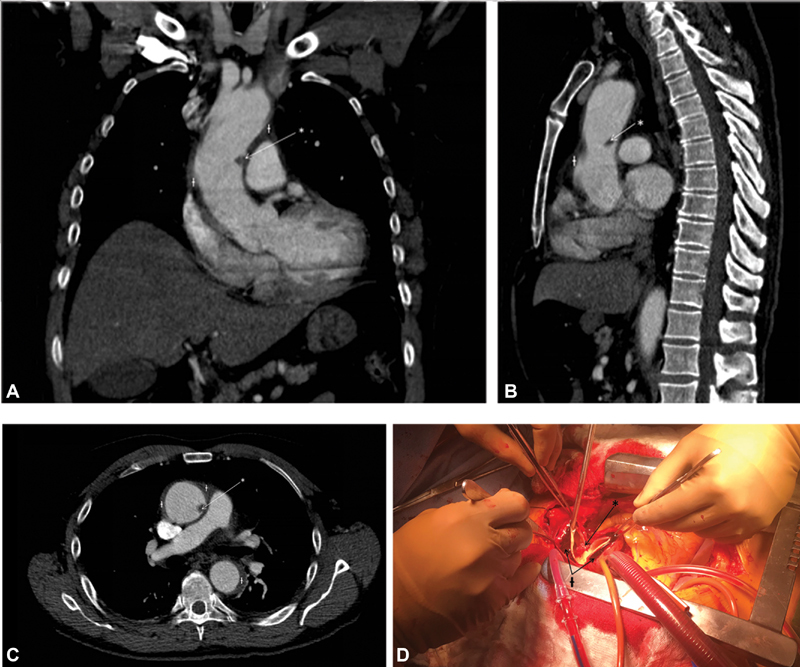
( **A–C** ) Chest computed tomography angiography revealing an aortic intramural hematoma in intraluminal filling defect. ( **D** ) Intraoperative thrombus covering a small intimal tea.

An ascending aortic replacement was performed under deep hypothermic circulatory arrest. A thrombus covering a small intimal tear was found within the aortic lumen at the distal ascending aorta corresponding to the filling defect ( Fig. 1D ). No other intimal tear was found. The hematoma was removed ( Fig. 1D ) to ensure secure proximal and distal anastomoses, no embolization occurred. The patient was discharged on postoperative day 6 without complication.

Disruption of a vasa vasorum within the media or bleeding within the vicinity of a penetrating atherosclerotic ulcer are the mechanisms reported to cause IMH. 1 2 Minimal intimal tear with secondary thrombus formation is the probable etiology of IMH in this case. Surgeons performing open and endovascular surgical repair should be aware of this finding and its risks for thromboembolic complications.
